# Effect of Low Environmental Pressure on Sintering Behavior of NASICON-Type Li_1.3_Al_0.3_Ti_1.7_(PO_4_)_3_ Solid Electrolytes: An *In Situ* ESEM Study

**DOI:** 10.1021/acs.cgd.2c01098

**Published:** 2023-02-17

**Authors:** Osmane Camara, Qi Xu, Junbeom Park, Shicheng Yu, Xin Lu, Krzysztof Dzieciol, Roland Schierholz, Hermann Tempel, Hans Kungl, Chandramohan George, Joachim Mayer, Shibabrata Basak, Rüdiger-A. Eichel

**Affiliations:** † Forschungszentrum Jülich GmbH, Institute of Energy and Climate Research—Fundamental Electrochemistry (IEK−9), 52428 Jülich, Germany; ‡ Dyson School of Design Engineering, Imperial College London, SW7 2AZ London, United Kingdom; § Ernst Ruska-Centre for Microscopy and Spectroscopy with Electrons and Peter Grünberg Institute, Forschungszentrum Jülich GmbH, 52428 Jülich, Germany; ∥ Central Facility for Electron Microscopy (GFE), RWTH Aachen University, 52074 Aachen, Germany; ⊥ Institute of Physical Chemistry, RWTH Aachen University, D-52074 Aachen, Germany

## Abstract

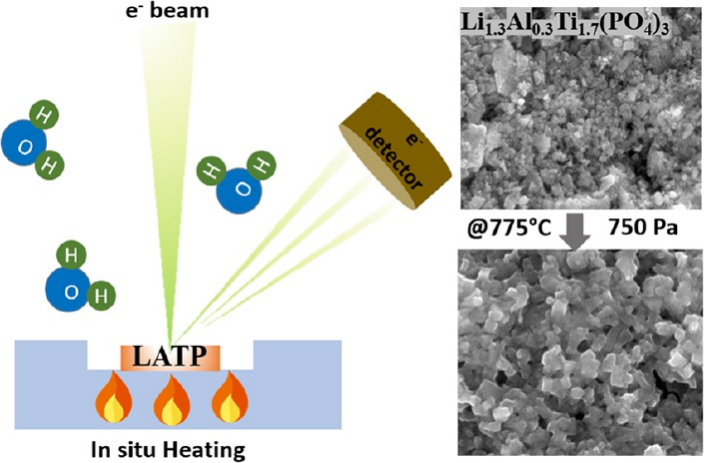

Solid-state sintering at high temperatures is commonly used to densify solid electrolytes. Yet, optimizing phase purity, structure, and grain sizes of solid electrolytes is challenging due to the lack of understanding of relevant processes during sintering. Here, we use an *in situ* environmental scanning electron microscopy (ESEM) to monitor the sintering behavior of NASICON-type Li_1.3_Al_0.3_Ti_1.7_(PO_4_)_3_ (LATP) at low environmental pressures. Our results show that while no major morphological changes are observed at 10^–2^ Pa and only coarsening is induced at 10 Pa, environmental pressures of 300 and 750 Pa lead to the formation of typically sintered LATP electrolytes. Furthermore, the use of pressure as an additional parameter in sintering allows the grain size and shape of electrolyte particles to be controlled.

## Abstract 2

Using environmental SEM with a heating stage, we probe the sintering behavior of Li_1.3_Al_0.3_Ti_1.7_(PO_4_)_3_ electrolytes *in situ*. We show that beyond a threshold pressure, sintering of the electrolyte starts to take place, and sintering at a pressure of 300 Pa or above leads to similar grain morphology as obtained at ambient atmosphere.

## Introduction

Recently, developing suitable solid-state electrolytes for solid-state batteries^1^ has been an important task because these electrolytes are safer and support high-energy electrodes. Therefore, all-solid-state batteries can better enable many applications, including in the automotive, electronic, aeronautic, and photovoltaic sectors.^2,3^ The NASICON-structured solid electrolyte Li_1.3_Al_0.3_Ti_1.7_(PO_4_)_3_ (LATP) is recognized to be one of the promising electrolytes due to their relatively high ionic grain conductivity, low toxicity, and good electrochemical stability while being easy to process and inexpensive.^2,4−8^ Similar to most solid-state electrolytes, LATP can be processed via solid-state sintering.^2^ Typically, pre-sintered materials are prepared by mixing powders and compacting them into a pellet to form the green compact, which is then sintered.

In the literature, the influence of the temperature on the properties (e.g., density, grain size, hardness) of LATP sintered under atmospheric pressure^3,10^ is a parameter being extensively investigated. Yan et al. have, for instance, reported that a temperature of 1100 °C was more suitable for the formation of dense enough electrolytes, while other authors such as Xu et al. have focused on the possibility of performing sintering at low temperatures.^7,10^ For this purpose, the authors proposed to use Li-rich green compacts, as the formation of a Li-rich liquid or glassy phase during reactive sintering led to a higher densification and formation of larger grains at temperatures as low as 775 °C. The ionic conductivity of the LATP electrolyte was reported to depend on how large the grains were after sintering, as bigger grains meant fewer grain boundaries and, thus, fewer regions with poorer ionic conductivity.^10^ While both the temperature and the nature of the green compact are known to play a role in the sintering process, the use of low environmental pressure during the sintering of electrolytes as a route is not currently explored. However, knowing how these conditions might affect the sintering process is crucial. In most studies, *ex situ* scanning electron microscopy (SEM) observations are performed to understand how the features of green compact such as particle size, shape, and distribution impact densification.^7,9^ As such observations are mostly made post-sintering, crucial information regarding the evolution of green compact is lacking, and it is therefore difficult to know which features of the sintering process or green compact need to be optimized. Indeed, as the exploration of sintering via *in situ* electron microscopy cannot be readily performed at atmospheric pressure at least in an open-cell configuration, it is of paramount importance to know if the sintering process can be induced under the vacuum of the microscope (≈10^–2^ Pa in the current work) and/or at the low environmental pressures available in environmental electron microscopes (maximum 750 Pa in the current work). Besides, manipulating the sintering environmental pressure may become an additional way of controlling the sintering rate and the nature of the sintered material. Indeed, important features of sintered materials such as hardness, grain size distribution, density, pores, or phase purity can be improved by performing sintering under these conditions as reported previously.^11−14^ For instance, vacuum sintering is often used as a way of increasing the phase purity of the sintered material via the evaporation of solvents or other impurities.^13−15^ Furthermore, such a method has also been used to control the grain size distribution in the sintered material.^14^ Vacuum or low environmental pressure sintering, therefore, provides an additional way of influencing the properties of sintered materials while being a method already implemented on a large scale in the industry (even though not yet on electrolytes).

With the democratization of environmental SEM (ESEM) and the introduction of devices dedicated to performing *in situ* heating, samples can be observed directly during sintering and under various atmospheres, environmental pressures, and temperatures.^16^ In this work, the above combination of ESEM coupled with a heating stage is used to sinter the green compacts of LATP samples similar to those used by Xu et al.^7^ at 775 °C under ambient atmosphere (at atmospheric pressure). Comparing the results of *in situ* heating experiments with those performed under an ambient atmosphere, the environmental pressures under which sintering can be induced are revealed. Furthermore, the way pressure can be used to control the sintering process is discussed by performing *in situ* sintering between 10^–2^ and 750 Pa.

## Experimental Section

Green compacts were prepared following the same method as Xu et al. to achieve low-temperature sintering of LATP.^7^ In short, all precursors were mixed in stoichiometric amounts, except for Li_2_CO_3_, which represented an excess of 10 wt % in the mix, whose purpose was to improve densification. Further details about the synthesis and the green compact are available in previous work.^7^

The green compact specimens were heated at 775 °C *ex situ* and *in situ* using an FEI heating stage, which allows the specimen to be heated to a maximum of 1000 °C. To induce sintering, the specimens were heated to 775 °C with a temperature ramp of 50 °C per minute and observed using an FEI Quanta FEG 650 in vacuum (≈10^–2^ Pa) and in ESEM mode. More details about the furnace and the *in situ* setup can be found in the literature.^16^ Experiments in ESEM mode were carried out under a water vapor environment at pressures ranging from 10 to 750 Pa (which are the minimum and maximum environmental pressures allowed under these conditions). In ESEM mode, the specimens were imaged using an acceleration voltage of 20 kV. To avoid charging, the microscope was operated at 2 kV under vacuum using the standard Everhart–Thornley detector while energy-dispersive X-ray spectroscopy (EDX) measurements were performed at 5 kV to allow emission of the characteristic X-rays.

The phase compositions of both the sintered and unsintered specimens were analyzed via an X-ray diffractometer (EMPYREAN, Panalytical) using Cu Kα radiation and a range of 2θ between 10 and 80° at 40 kV with a step size of 24.77 s/0.0084° at 40 mA. To highlight the morphology changes during sintering via edge detection, ESEM images were processed by the Canny edge detection method (σ = 2) with the Scikit Image python package.^17,18^

## Results and Discussion

**Figure 1:**
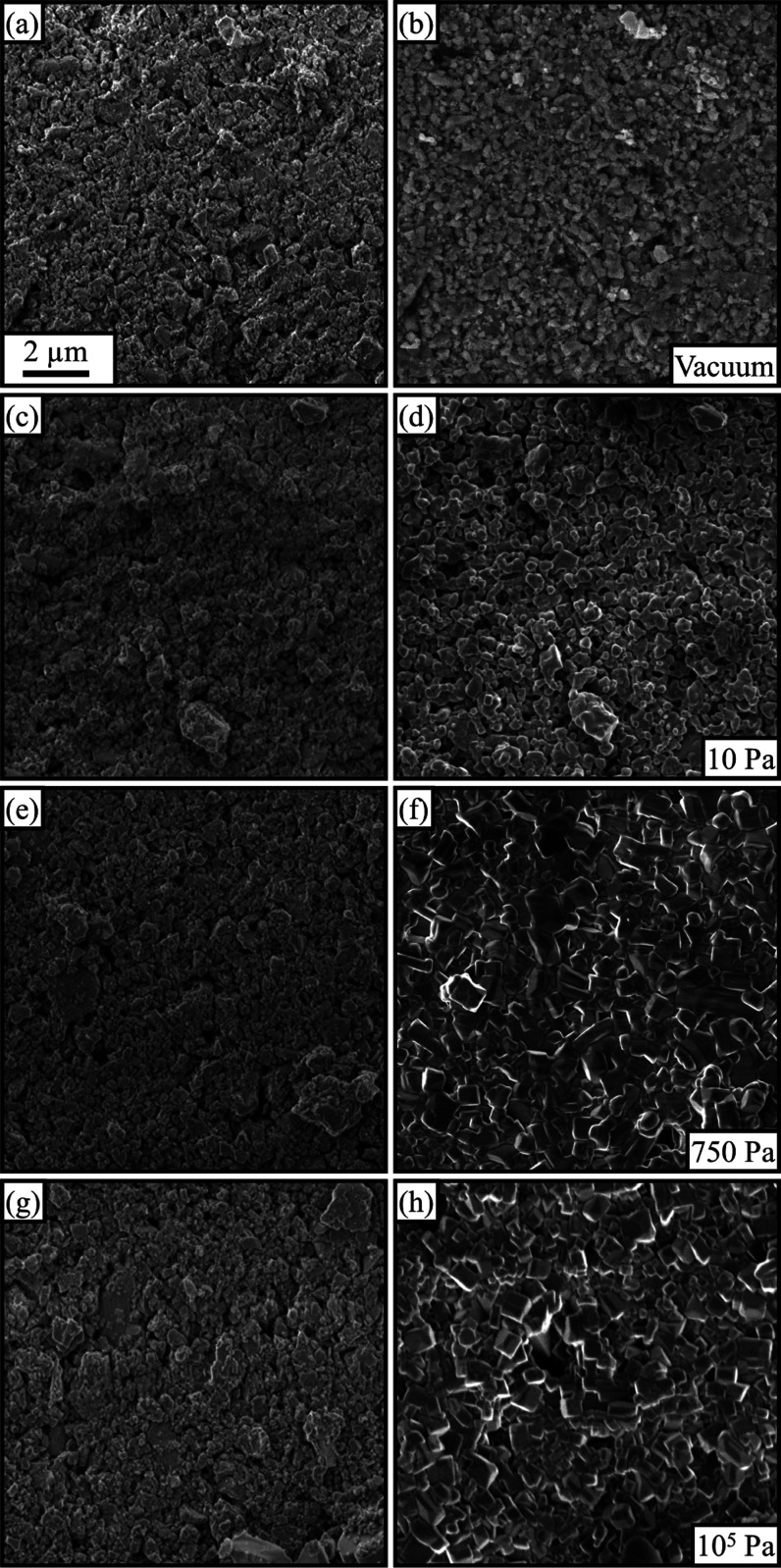
Left column shows representative SEM images of the green compact electrolyte (a, c, e, g) before heating. The SEM images in the right column show the same region of the specimens after heating at 775 °C for 2 h. The images were obtained after heating (b) under vacuum (≈10^–2^ Pa), at (d) 10 Pa, (f) 750 Pa, and (h) at atmospheric pressure (≈10^5^ Pa). The heating at 10^–2^, 10, and 750 Pa is performed *in situ*, and the heating at atmospheric pressure is performed outside the SEM using the same heating device. The scale bar in panel (a) applies to all images.

To determine if there are conditions under which sintering can be successfully performed *in situ*, the Li-excess intermediate LATP green compacts were heated at 775 °C for 2 h under different environmental pressures, namely, under vacuum (≈10^–2^ Pa) and at 10, 300, and 750 Pa inside the ESEM. For comparison, a green compact specimen was heated at 775 °C using the same heating device under an ambient atmosphere, as such conditions are known to induce reactive sintering and densification. To verify whether the environmental pressure during heating influenced the elemental composition of the specimens, EDX measurements were performed. Results showed (Table S1 in the Supporting Information) that the pressure did not play any major role in terms of the sample elemental composition. Further, the elemental composition was homogeneous within different areas of the heated specimen. Using X-ray diffraction (XRD), it was furthermore confirmed that after the 2 h of heating, unlike in the pristine material, which contains a large amount of precursors and intermediate phases (as shown in our previous work),^7^ the crystalline phase was nearly pure LATP in all specimens (see Figure S1). Major differences between the various heating conditions were further revealed when observing the morphology of the specimens. Figure 1 shows representative SEM images before and after heating at different pressures (and a larger field of view of the samples after heating is also shown in Figure S2). The specimen heated under vacuum showed no visible morphological changes (Figure 1a,b). The heating under 10 Pa (Figure 1d) resulted in considerable grain growth and the presence of large pores. It furthermore led to the formation of particles having a morphology far from the rectangular or cubic shape usually induced when sintering is performed under an ambient atmosphere, as shown in Figure 1h.^7^ On the other hand, after heating at 750 and 300 Pa (Figures 1f and S3), the specimens were densified, and the typical rectangular-shaped particles were observed as being similar to the specimen heated under ambient atmosphere. Considering both the chemical analysis and the SEM observation, it can be stated that the grain growth was effectively induced at 300 Pa and above. On the other hand, heating performed under vacuum resulted in chemical reactions (in the course of which the precursors turned into LATP) but not in grain growth. Such observations indicate that the monitoring of the sintering process is only made possible if the microscope can be operated above a threshold environmental pressure.

**Figure 2:**
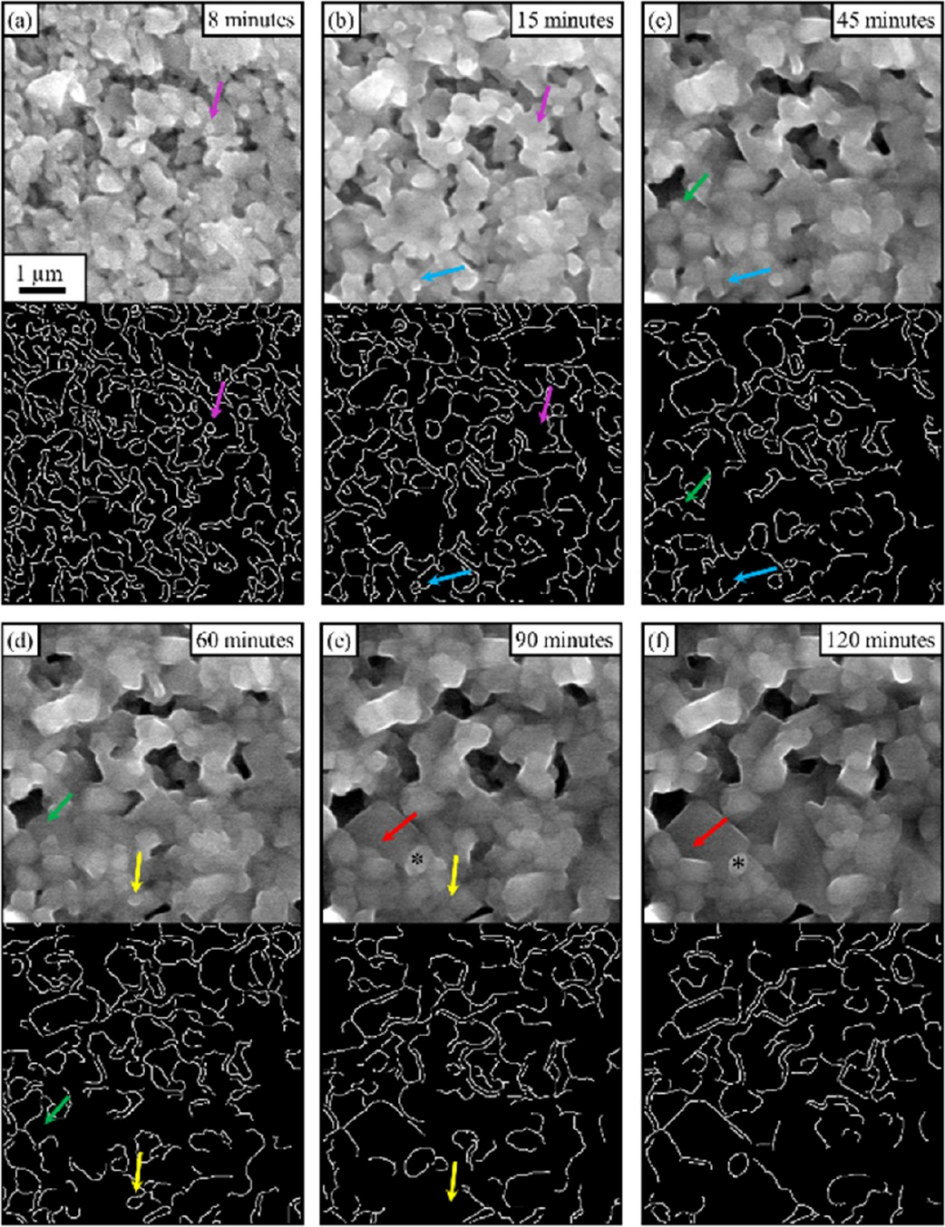
ESEM images showing the typical example of the grain morphology changes during *in situ* sintering of the LATP green compact at 750 Pa and 775 °C. For each image, edge detection was performed using the Canny edge method. The evolution of a few selected grains is indicated by colored arrows. To show an example of the volume reduction of grains, one grain is highlighted using an asterisk. The scale bar in (a–f) applies to all images.

As evidenced by Figure 1, substantial changes (i.e., formation of cubic-like grains and densification) occurred to the sample during the 2 h of heating at 750 Pa. One of the crucial changes expected during sintering is the elimination of pores, as it is associated with densification. As illustrated in Figure 2, the grains of the LATP specimen during the *in situ* heating are progressively merging to form larger grains. To better visualize this, a few grains are highlighted in the figure using colored arrows and an image generated via Canny edge detection.^17,18^ While the detection does not exactly follow all of the grains, it allows a qualitative visualization of the number of grains, which are shown to progressively decrease during sintering. Furthermore, it is observed that the elimination of grains depends on their size, i.e., small grains tend to be eliminated faster than larger ones. For instance, the grain indicated by the purple arrow and measuring 0.03 μm^2^ in Figure 2a is eliminated within 7 min (Figure 2b), while the grain indicated by the black star in Figure 2e,f progressively shrinks from 0.29 to 0.14 μm^2^ in 30 min. Hence, as larger grains are less rapidly eliminated, the morphological changes become less and less evident. The monitoring of the specimen in ESEM mode is further shown in Movie S1 and Figure S4. These also show that the elimination of pores and interparticle voids is predominant during the first 15 min of sintering while being slower afterward. Such rapid transformation occurring during the first phase of the sintering is in agreement with the literature, as dilatometry measurements show that at atmospheric pressure, densification of LATP also occurs at a faster rate at the beginning of the sintering process.^7^ It can be proposed that although the *in situ* observations are made at lower pressure in the current work, they are suited to describe the standard sintering process.

While it is necessary to readjust the ESEM and compensate for a large thermal drift during the first few minutes of the sintering, the current setup readily allows imaging a given area of the specimen before and after heating. This can be done by simply switching the ESEM to vacuum mode without retrieving the specimen, thus allowing an accurate evaluation of the early stages of the sintering process. A given region of a specimen was thus imaged in vacuum mode before and after a short heating time. Green compact specimens were heated in ESEM mode at 775 °C and 750 Pa, but the heating was either stopped as soon as the furnace reached a temperature of 775 °C (Figure S5) or after 5 min at 775 °C (Figure S6). The comparison between the initial and the final state of the heated specimens showed that during the thermal ramp, the specimen remained unchanged. It must however be noted that just after the thermal ramp (i.e., at 0 min at 775 °C), the temperature of the specimen may be lower than 775 °C due to the relatively fast heating rate. After just 5 min into the sintering, major changes occurred to the specimen, further confirming that even at 750 Pa, densification starts at an early stage of sintering but not during the thermal ramp.^7,19^ Small cubic-like grains with an average size of 0.07 μm^2^ were formed, and pores and particles which were smaller than ≈0.02 μm^2^ that were initially present in the green compact were no more (Figure S7). On the contrary, pores larger than 0.2 μm^2^ remained after the 5 min heating process (Figure S6). In this work, it was observed that such large pores in the green compact tended to be stable during the 2 h of *in situ* heating. It must be noted that in the literature, even after a longer sintering time, the presence of pores in sintered electrolyte materials remains an issue.^7,9^ It must be noted that the 5 min of heating did not only cause smaller particles to merge, as particles larger than 1 μm^2^ started to dissociate into smaller ones (see Figure S6). This contrasts with the expected Ostwald ripening behavior, where large particles would be expected to grow at the expense of smaller ones.^20^ Yet, the morphological changes observed here can be more complex as they co-occur with chemical reactions. In any case, as both the population of the smallest and largest particles lessens, the sintering leads to a homogenization of the grain size during the early stage of the process.

**Figure 3:**
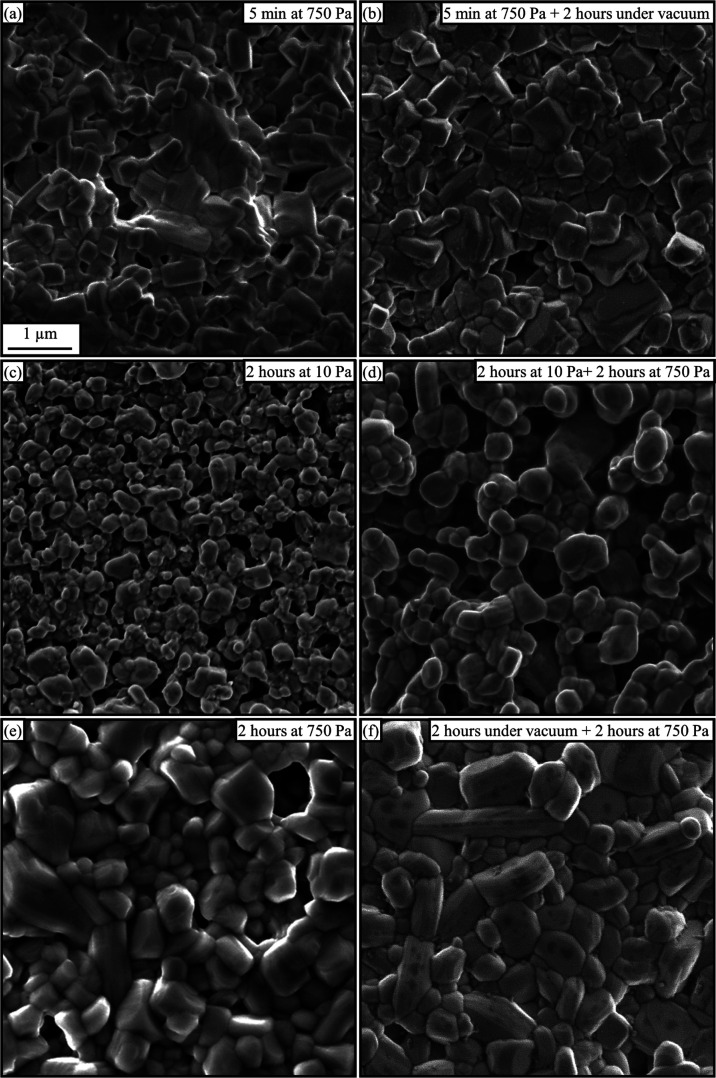
Representative SEM images of LATP specimens recorded after heating at 775 °C (a) for 5 min at 750 Pa; (b) for 5 min at 750 Pa, followed by 2 h under vacuum (≈10^–2^ Pa); (c) for 2 h at 10 Pa; (d) for 2 h at 10 Pa, followed by 2 h at 750 Pa; (e) for 2 h at 750 Pa; and (f) for 2 h under vacuum (≈10^–2^ Pa), followed by 2 h at 750 Pa. The scale bar in (a) applies to all images.

To evaluate if the environmental pressure can be used to dynamically influence the sintering, a specimen was heated at 750 Pa for 5 min, after which the ESEM was switched into vacuum mode for 2 h while maintaining the same temperature (i.e., 775 °C). The specimen heated solely at 750 Pa for 5 min is shown in Figure 3a, and the sample heated at 750 Pa for 5 min, followed by 2 h in vacuum, is shown in Figure 3b. Both the samples exhibited relatively small grains with similar average grain size (*A*). This shows that grain size does not solely depend on the heating time (*A* ≈ 0.06 μm^2^ after 2 h and 5 min and *A* ≈ 0.07 μm^2^ after 5 min). In comparison, specimens heated for 2 h at 750 Pa (Figure 3e) had much larger grains (*A* ≈ 0.14 μm^2^). Therefore, it can be proposed that even though the electrolyte shown in Figure 3b was heated for 2 h and 5 min, the sintering process was interrupted when the ESEM was switched into vacuum mode. This evidenced that although the usual way of controlling the sintering process is to vary the temperature, the environmental pressure can further be used to dynamically control it. Additionally, we compare a green compact that was heated at 10 Pa for 2 h (Figure 3c) with a specimen that was first heated for 2 h at 10 Pa, followed by heating at the pressure of 750 Pa for two additional hours (Figure 3d). As shown in Figure 3d, after 4 h of heating, the interconnectivity of the grains in the specimen was similar to that of a specimen only heated at 10 Pa. In fact, even though the grains were substantially larger after the 4 h of heat treatment (i.e., *A* ≈ 0.07 μm^2^ after 4 h and *A* ≈ 0.02 μm^2^ after 2 h at 10 Pa), in both cases, numerous pores were observed, as well as a network of coarse grains without the aforementioned cubic-like shape. It could have been argued that the morphology induced after the 10 Pa heating was due to a slower sintering rate. However, if using an environmental pressure of 10 Pa simply caused a decrease in the sintering rate, the specimen would have experienced major changes during the subsequent 750 Pa heating and exhibited the typical morphology shown in Figure 3e (LATP specimen heated for 2 h in 750 Pa). On the other hand, when a green compact was successively heated for 2 h under vacuum and 2 h at 750 Pa (Figure 3f), the second heating step at 750 Pa caused the formation of the usual cubic-like grains (with *A* ≈ 0.14 μm^2^) and the removal of pores. As stated above, after heating under vacuum for 2 h, there are almost no precursors and intermediate phases left in the specimens. Such chemical changes do not normally prevent sintering from taking place when the pressure is increased. Likewise, as the chemical composition of the specimen at 10 Pa is similar to that of the vacuum one, its chemical composition should also allow sintering during subsequent heating at 750 Pa. It can be concluded that it is the morphology of the 10 Pa specimens which prevents densification to occur through the subsequent heating at 750 Pa. During the heating of a green compact, the main driving force behind sintering is the diminution of surface energy, which will cause coarsening (where the grains become larger) or densification (where pores are closing, and the shape of the grains may lose their sphere-like shape and become more cubic). For the 10 Pa specimen, the SEM images evidenced that coarsening was the main effect of the heating process, hence, showing that the way the material diffuses during heating can be controlled by the environmental pressure. Furthermore, while the pristine green compacts are made of small particles and small pores to facilitate diffusion between adjacent grains, after heating under 10 Pa, the specimen is, on the contrary, made of coarse grains and larger pores. For such morphology, heating will favor coarsening instead of densification.^21^

The average grain size of the LATP green compact sintered at 300, 750 Pa, and in air was measured to be 0.18, 0.14, and 0.11 μm^2^, respectively. The measurements thus revealed that under conditions favorable for sintering, the lower the environmental pressure, the bigger the grains are. This is clearly evidenced in Figure S8, as the grains formed under ambient atmosphere are visibly smaller than the grains formed at 300 Pa. In LATP electrolyte, grain boundary conductivity limits the bulk ionic conductivity. Thus, it is believed larger grain size should lead to higher ionic conductivity.^7^ Our results show that low-pressure sintering can be used to control the grain sizes, and thus, suitable use of low-pressure sintering can enhance the electrolyte ionic properties.

At the beginning of the sintering process, evaporation–condensation (E–C) is considered to be the main mechanism behind morphological changes in submicron ceramic powders.^22^ Furthermore, this mechanism can be favored at low environmental pressures as evaporation becomes more likely.^15,23^ In this work, it is therefore reasonable to assume that E–C becomes more likely to occur when the environmental pressure is low. It is important to recall that the E–C process is known to induce larger grains.^15,20^ For these reasons, the lower pressure can reinforce E–C and contribute to the formation of the larger grains observed here at 300 and 750 Pa as compared with those induced at atmospheric pressure. Typically, grain growth and densification are interdependent during sintering. If a mechanism leading to mass transport from the surface to the neck of two particles (e.g., via E–C, surface diffusion, and volume diffusion) is favored, coarsening will occur more readily while densification processes may decrease as the overall surface area of grains is lessened due to grain growth.^20,24,25^ Hence, if the E–C contribution to the sintering process becomes too important, coarsening will become a major outcome of sintering and will hinder densification. This is a valid reason to elucidate why only coarsening is observed at 10 Pa.

The presence of impurities can also play a role in the difference in grain sizes. In practice, when small grains are desired, impurities can even be voluntarily introduced to retard grain growth, as previously reported.^26^ Indeed, it is known that impurities within the green compact can cause the pinning of grain boundaries during sintering. Such pinning will decrease the grain boundary mobility and thus result in smaller grains.^26^ Numerous works have shown that the volatilization of impurities was more pronounced when sintering was performed at low environmental pressure.^13−15^ Here, evaporation of impurities may therefore be facilitated within the ESEM. The evaporation of pinned impurities will thus free the grain boundaries and may therefore lead to larger grains. On the other hand, it must be noted that volatilization could also prevent sintering from occurring when the vaporized species belong to the material which should be sintered. As shown in the literature,^15^ evaporation of chemical elements during sintering is a commonly known issue that becomes more pronounced at low environmental pressures. In the LATP specimens used in this work, this issue may become particularly problematic under vacuum (i.e., 10^–2^ Pa) and thus prevent sintering.

In the specimens used here, the formation of Li-rich glassy phases via melting during sintering was reported in the work of Xu et al.^7^ As stated above, the authors reported that the glassy phase may have been responsible for the formation of larger grains as such a phase can promote atomic diffusion.^27^ The features of the liquid phase can be influenced by the temperature at which the sintering is performed.^19,27^ The elevated temperature can cause the evaporation of the liquid phase, impeding the sintering process.^27,28^ In contrast, the elevated temperature can also facilitate the melting of a solid phase into a liquid (or glassy-like) phase.^27,28^ In such instances, the elevated temperature will favor liquid phase sintering. Similarly, a lower environmental pressure during sintering could play an identical role as melting will occur more readily. Consequently, the formation of larger grains at 750 and 300 Pa can also be the result of an increase in the Li-rich glassy phase at low pressures.

**Figure 4:**
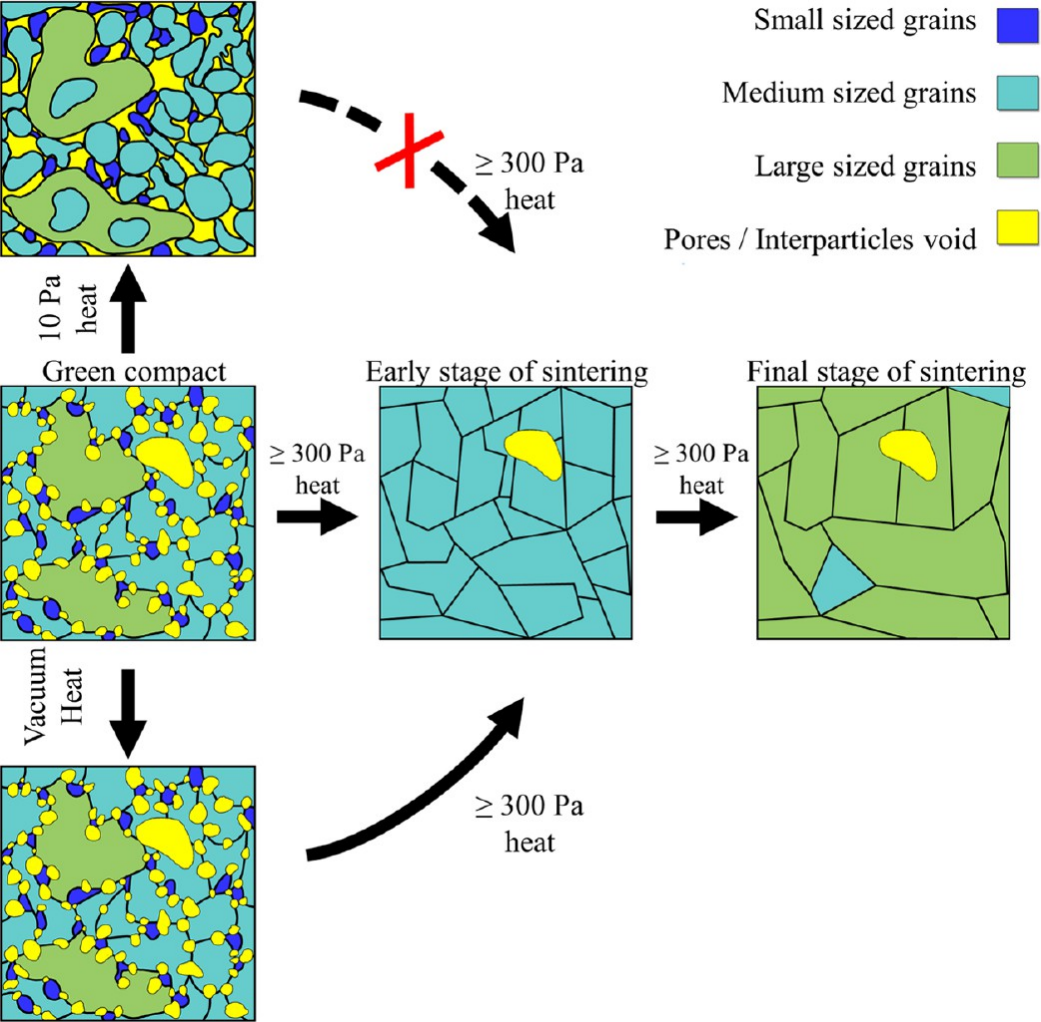
Schematics of the structural evolution of LATP green compact during sintering depending on the environmental pressure. The dashed black arrow with a red cross indicates a forbidden path.

The structural evolution of LATP green compacts can be summarized as follows and by the schematic in Figure 4. Above 300 Pa, rapid densification occurs at an early stage as most pores (shown in yellow in the figure) disappear at the beginning of the sintering process, and grains take upon a cubic-like shape. Likewise, the smallest particles (shown in blue) disappear while the largest (green) decompose into smaller ones, thus leading to a more uniform grain size distribution. At 775 °C and 750 Pa, these smallest grains are those whose sizes are below 0.02 μm^2,^ and the largest ones, those with grain sizes above 1 μm^2^. As described by the schematic, while most pores rapidly vanished, the largest ones (i.e., ≥0.2 μm^2^ at 750 Pa and 775 °C) tended to remain stable during sintering at such pressures. Furthermore, as stated above, the environmental pressure can be adjusted to induce the formation of larger grains within the sintered material or induce more drastic effects on the specimen morphology. For instance, as shown in the schematic, at 10 Pa, only coarsening is induced, and densification cannot be performed even by subsequently raising the environmental pressure ≥300 Pa. On the other hand, performing the heating under vacuum can halt the sintering while allowing the morphological transformation to proceed when the environmental pressure is raised above (e.g., ≥300 Pa at 775 °C).

## Conclusions

Using an ESEM coupled with a heating device, we have collected key qualitative and quantitative information about NASICON-type solid electrolyte sintering and densification behavior. Densification during sintering was shown to only be possible above a threshold environmental pressure, thus demonstrating the practicality of environmental electron microscopes for *in situ* processing. While this threshold environmental pressure will depend on the nature of the materials as well as on the sintering temperature, the low pressure itself can influence some features of the sintering and induce the formation of larger grains, larger pores, grains with a different shape, or the volatilization of impurities. Lastly, this work shows strong evidence that environmental pressure can be used as a way of interrupting or modifying the sintering and grain growth of LATP.

## Supporting Information Available

XRD and SEM images further demonstrate the sintering behavior of the LATP green compact at different conditions (PDF)
Typical sintering process of LATP green compact (Movie S1) (AVI)

## Supplementary Material

cg2c01098_si_001.pdf

cg2c01098_si_002.avi
