# Supplementary material for: Effect of Low Environmental Pressure on Sintering Behavior of NASICON-Type Li1.3Al0.3Ti1.7(PO4)3 Solid Electrolytes: An In Situ ESEM Study
Source: Cryst Growth Des. 2023 Feb 17;23(3):1522–9. doi: 10.1021/acs.cgd.2c01098 (PMC9983001; doi:10.1021/acs.cgd.2c01098)
Supplement: Supplementary file 1 — cg2c01098_si_001.pdf [file cg2c01098_si_001.pdf]

Supporting information

Effect of Low Environmental Pressure on Sintering Behaviour of NASICON-type $Li_{1.3}Al_{0.3}Ti_{1.7}(PO_4)_3$ Solid Electrolytes: An In-situ ESEM Study

Osmane Camara[1,±], Qi Xu[1], Junbeom Park[1], Shicheng Yu[1], Xin Lu[1], Krzysztof Dzieciol[1], Roland Schierholz[1], Hermann Tempel[1], Hans Kungl[1], Chandramohan George[2], Joachim Mayer[3,4], Shibabrata Basak[1,3,*], Rüdiger-A. Eichel[1,5]

[1]Forschungszentrum Jülich GmbH, Institute of Energy and Climate Research – Fundamental Electrochemistry (IEK–9), 52428 Jülich, Germany

[2]Dyson School of Design Engineering, Imperial College London, SW7 2AZ London, United Kingdom

[3]Ernst Ruska-Centre for Microscopy and Spectroscopy with Electrons and Peter Grünberg Institute, Forschungszentrum Jülich GmbH, 52428 Jülich, Germany

[4]Central Facility for Electron Microscopy (GFE), RWTH Aachen University, 52074 Aachen, Germany

[5]Institute of Physical Chemistry, RWTH Aachen University, D-52074 Aachen, Germany

± o.camara@fz-juelich.de

* s.basak@fz-juelich.de

Movie S1. Shows the LATP sintering process at 300 Pa at 775°C.

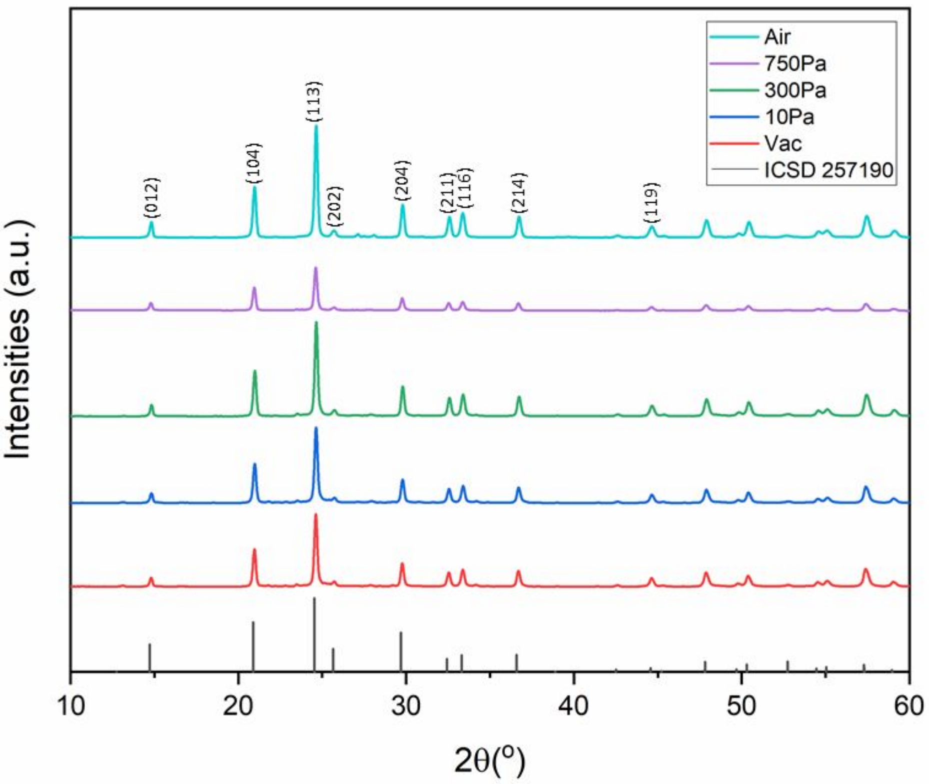

Figure S1. XRD patterns of the LATP green compact heated at 775°C at different environmental pressures. The pressure under vacuum is ≈ 10^{-2} Pa and the ISCD corresponds to the standard $Li_{1.3}Al_{0.3}Ti_{1.7}(PO_4)_3$.

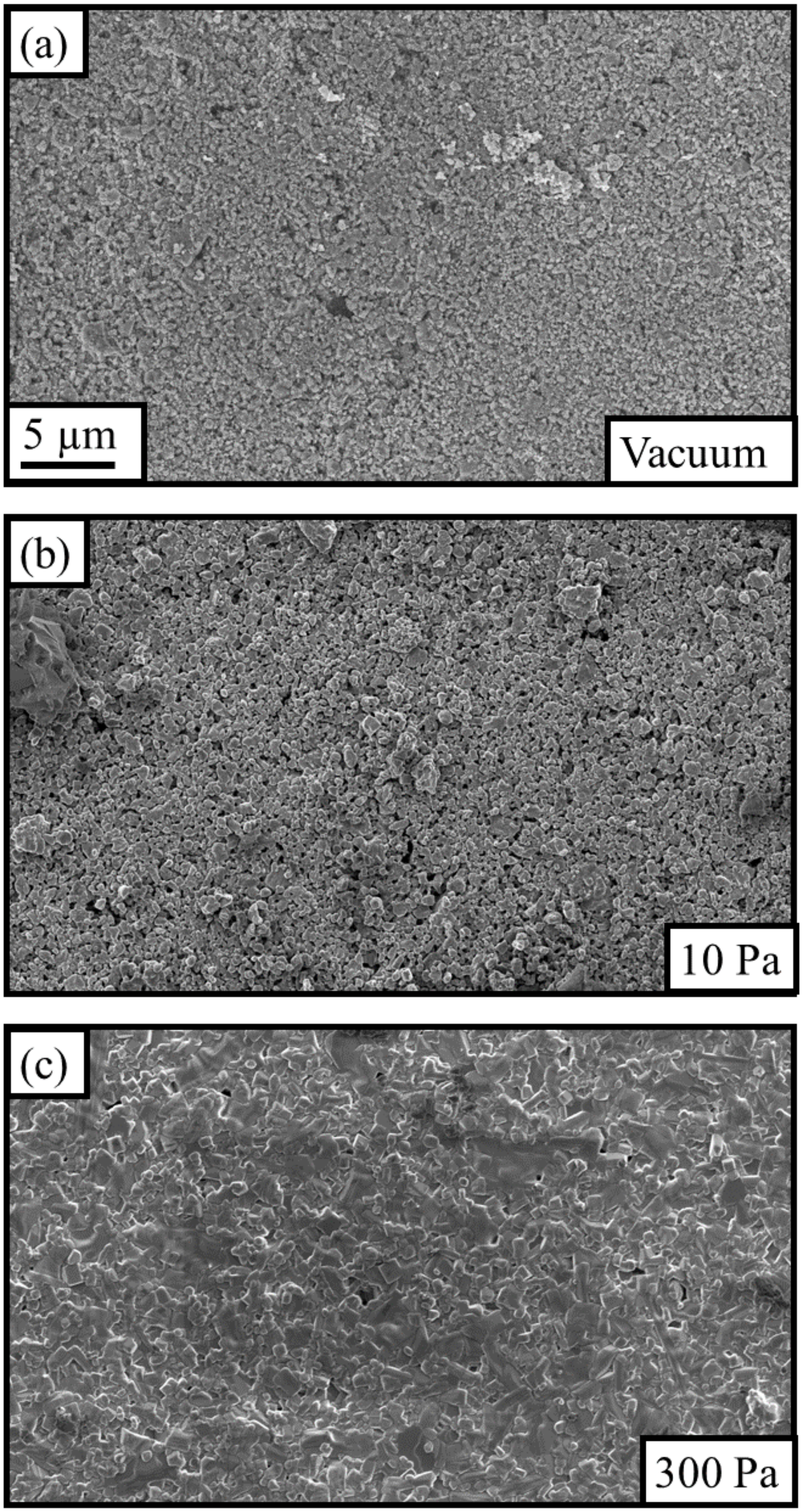

(a)
5 μm
Vacuum
(b)
10 Pa
(c)
300 Pa

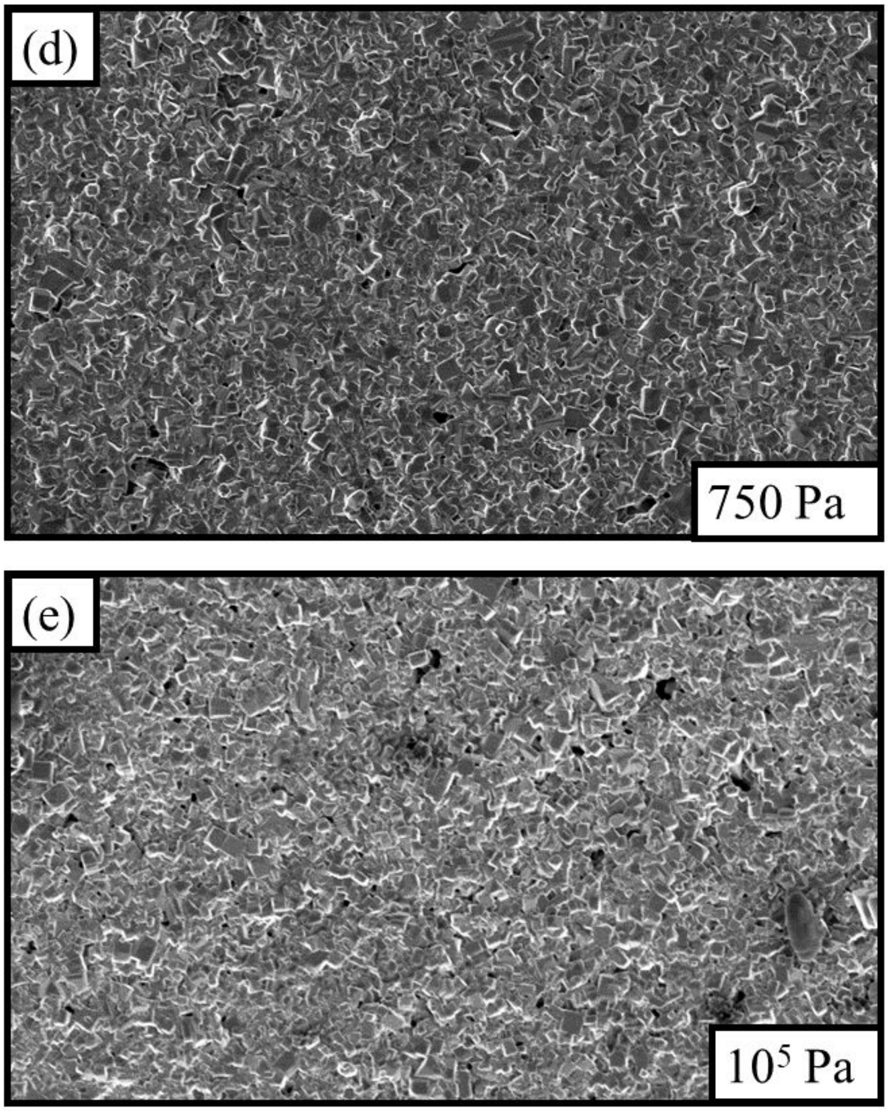

Figure S2. SEM images of the electrolyte after heating at 775°C for 2 hours at (a) 10^{-2} Pa, (b) 10 Pa, (c) 300 Pa, (d) 750 Pa and (e) 10^5 Pa. The scale bar in (a) applies to all images.

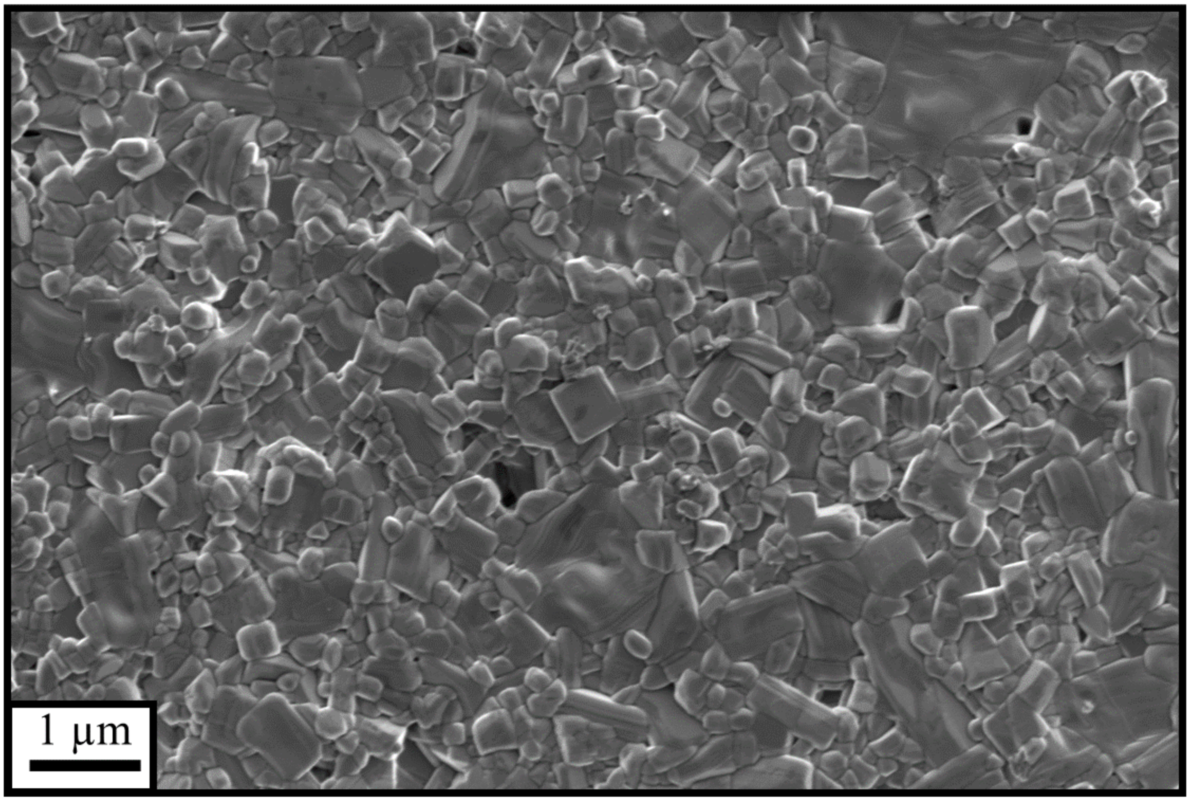

Figure S3. SEM image of the LATP specimen after heating for 2 hours at 775°C at 300 Pa at a higher magnification.

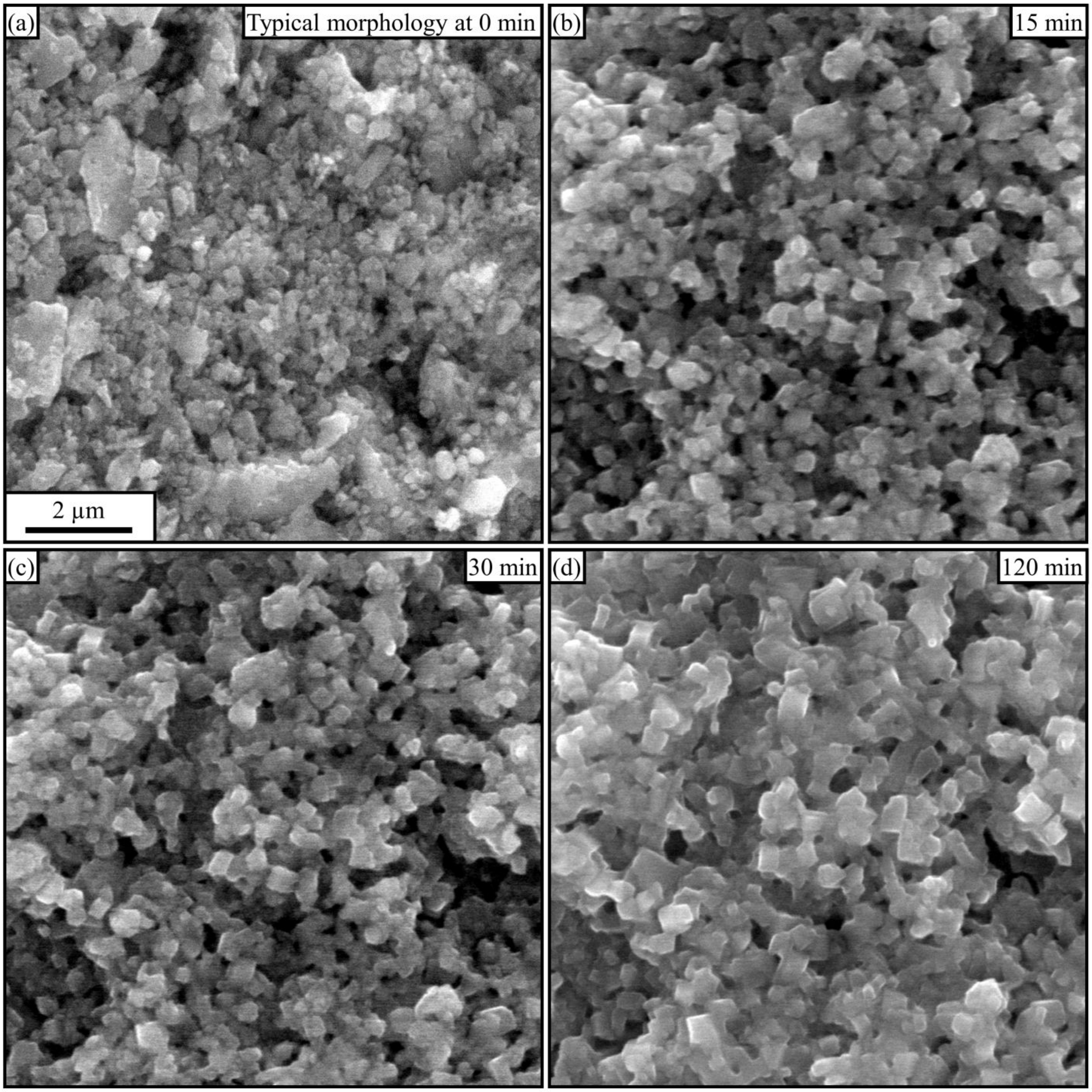

Figure S4. ESEM images of the LATP specimen (a) before sintering and (b) - (d) of a given area during sintering at 750 Pa. The scale bar in (a) applies to all images.

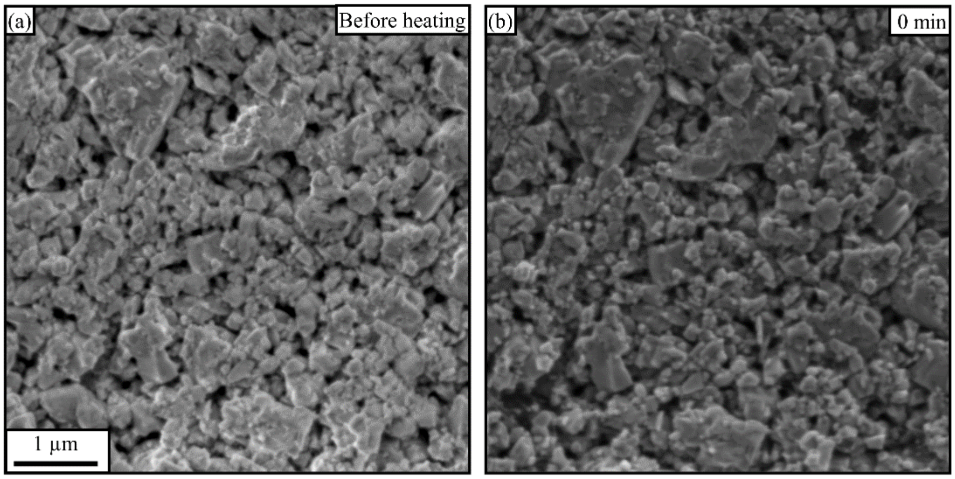

Figure S5. SEM images of a LATP specimen recorded (a) before heating and (b) directly after ramping to 775°C with a heating rate of 50°C/min at 750 Pa. The scale bar in (a) also applies to (b).

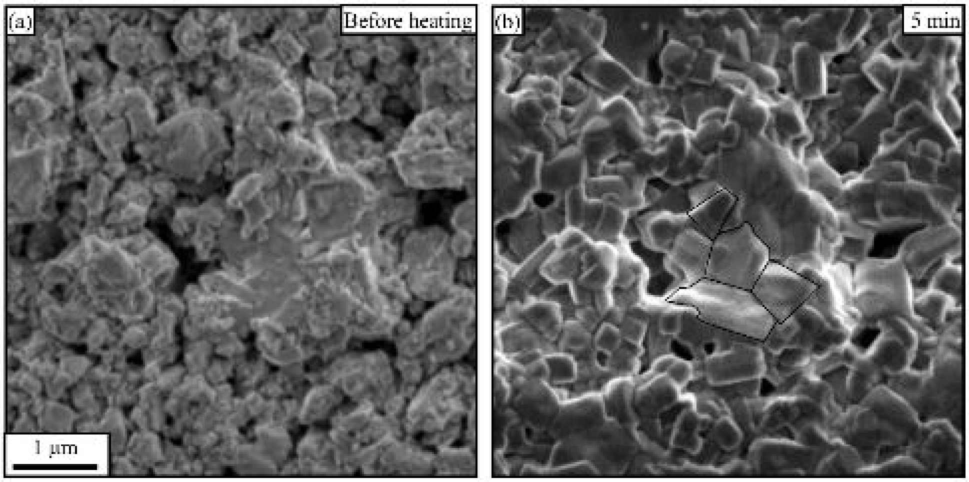

Figure S6. SEM images of a LATP specimen r (a) before heating and (b) after heating at 775°C for 5 minutes at 750 Pa. The scale bar in (a) also applies to (b). The black lines show an example of the dissociation of a large particle.

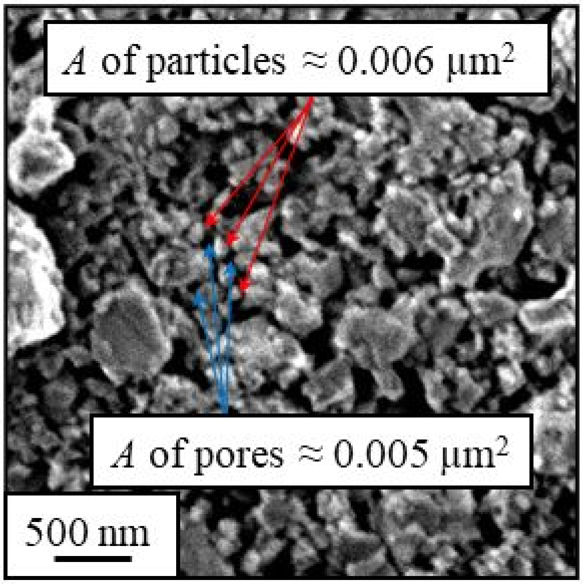

Figure S7. SEM images of a LATP green compact before sintering. Three submicron pores and particles are highlighted via blue and red arrows, respectively.

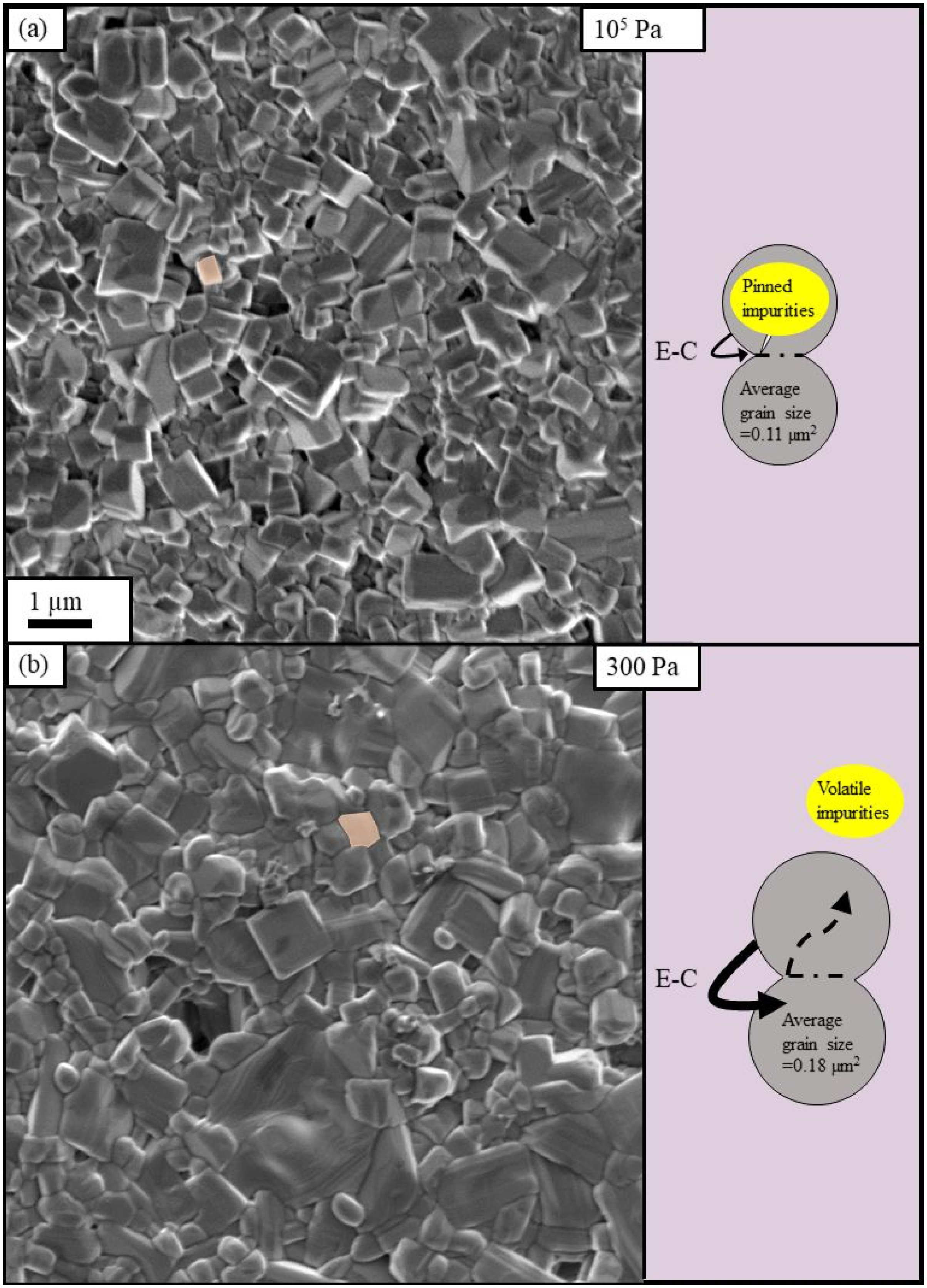
(a)
10^5 Pa
Pinned impurities
E-C
Average grain size =0.11 μm²
1 μm
(b)
300 Pa
Volatile impurities
E-C
Average grain size =0.18 μm²

Figure S8. SEM images and corresponding schematic representations of a LATP specimen after sintering at 775°C for 2 hours at (a) atmospheric pressure and (b) at 300 Pa. Highlighted grains in (a) and (b) represent the average grain size in each case. The scale bar applies to both SEM images. The schematics indicate that evaporation-condensation and evaporation of impurities are more pronounced at 300 Pa.

	**Elements**			
**Samples**	Titanium (%)	Oxygen (%)	Aluminum (%)	Phosphorus (%)
Pristine	11	67	2	19
Vacuum	12	67	2	18
10 Pa	11	67	3	20
300 Pa	12	68	2	18
750 Pa	11	68	2	18
Air	12	66	2	20

Table S1. Chemical composition of the LATP specimen before and after heating at 775°C for 2 hours at different pressures as measured using EDX in vacuum mode.
